# Editorial: Navigating the psychological landscape of remote work: understanding, resilience, and well-being, volume II

**DOI:** 10.3389/fpsyg.2025.1681363

**Published:** 2025-09-05

**Authors:** Naval Garg, Freda Van Der Walt, John Burgess

**Affiliations:** ^1^Delhi Technological University, New Delhi, India; ^2^Central University of Technology, Bloemfontein, South Africa; ^3^Torrens University Australia, Adelaide, SA, Australia

**Keywords:** remote work, working from home, working remotely, digital evolution, technostress

Remote work has traditionally been part of various sectors, including transportation, construction, mining, defense, education, and healthcare. In the past, working remotely required being physically separated from the office, frequently in hazardous circumstances. Informal industries such as textiles and agriculture continue to follow this model in emerging markets. However, due to the gig economy and digital platforms that allow for flexible work schedules and a variety of career models, remote work has significantly increased as a result of globalization, digitization, and widespread internet access.

Millions of people around the world were forced to work from home due to the COVID-19 pandemic, which served as a strong catalyst for remote work. This change questioned established work practices and brought attention to the advantages and disadvantages of working remotely. The hybrid work model, which divides time between the office and home, became a viable long-term solution as remote work grew in popularity.

Making the distinction between working from home and working remotely is essential. This volume focuses on home-based work made possible by digital tools that keep employees connected to central systems and teams, even though remote work can take place from a variety of locations.

Volume 2 aims to expand on our understanding of the psychological effects of working remotely, particularly in the context of digital evolution, workforce dynamics, and managerial frameworks. This volume includes five articles that apply survey-based approaches to explore these challenges across diverse cultural and occupational settings.

Buzás et al. investigate technostress and its effects on employees' voice behaviors in organizations. They identify challenge stressors, such as techno-uncertainty and techno-overload, which can enhance intrinsic motivation and affective commitment. Hindrance stressors such as techno-insecurity and techno-complexity, on the other hand, limit engagement. The study suggests that organizations can harness challenge stressors to foster positive voice behaviors if managed effectively.

Prasad et al. examine the link between organizational citizenship behavior (OCB) and psychological well-being, utilizing emotional intelligence as a mediating factor. A survey of 300 IT professionals in Hyderabad, India, indicates that emotional intelligence enhances the beneficial impact of OCB on well-being, which in turn improves performance. The authors recommend promoting emotional intelligence as a strategy to improve remote work dynamics.

Wan et al. focus on the well-being of gig workers in China. Their survey of 420 gig workers, based on cognitive-affective processing system framework, shows that job autonomy has a positive influence on workplace well-being, with work alienation and positive emotions acting as mediators. The study also shows that algorithmic control can have an effect on the results, suggesting that platforms can improve psychological support by giving workers more freedom at work.

Simon et al. examine the influence of technostress on employees' willingness to continue working from home. Their study of 361 office workers found that technostress alone does not significantly affect workers' preferences for remote work. Job satisfaction and employment status, however, are the mediating factors. The authors suggest certain approaches to reduce technostress while maintaining the flexibility of remote work.

Li et al. evaluate the influence of middle managers' digital leadership on engaging employees during the digital transformation process. Based on a sample of 559 respondents from Southwest China, the study concludes that empowerment and affective commitment mediate the relationship between digital leadership and engagement, with emotional intelligence acting as a moderator. The authors suggest that digital leadership competencies should be emphasized to foster stronger engagement in remote work settings.

Addressing the psychological health of remote workers is becoming more and more crucial as remote and hybrid work models continue to expand. The results in this volume sheds light on how leadership, job autonomy, emotional intelligence, and technostress affect remote work environments. As the nature of work continues to change, these studies provide organizations with useful advice on how to build more resilient, human-centered work environments.

